# Clinical and surgical outcomes of virginal breast hypertrophy (VBH) in adolescent females: a systematic review of case reports

**DOI:** 10.1097/MS9.0000000000003153

**Published:** 2025-03-19

**Authors:** Erum Siddiqui, Maliha Khalid, Muhammad Saad Khan, Umaimah Naeem, Aminath Waafira

**Affiliations:** a Department of Medicine, Jinnah Sindh Medical University, Karachi, Pakistan; b School of Medicine, The Maldives National University, Malé, Maldives

**Keywords:** case reports, juvenile breast hypertrophy, psychological impact, subcutaneous mastectomy, virginal breast hypertrophy

## Abstract

**Background:**

The rare medical condition known as Virginal Breast Hypertrophy causes massive adolescent breast growth that leads to the important health problems with social implications. This systematic review examines treatment procedures and psychological effects along with identifying remaining knowledge gaps regarding this disorder.

**Methods:**

The research team executed a PRISMA-based literature search covering cases from the year 1934 to 2023, and analyzed 69 cases of VBH. The analysis included information about symptoms at diagnosis and treatment methods along with the recurrence rates and details about psychological elements. The study included patients who experienced juvenile breast hypertrophy during adolescence.

**Results:**

VBH typically presented with rapid bilateral breast enlargement (91.3%) and psychological distress (71%). The mean age of onset was 11.27 years, with disease progression averaging 10 months. Menarche was documented in 43.75%. Subcutaneous mastectomy showed the lowest recurrence rate (5.8%), while reduction mammoplasty had a recurrence rate of 44.7%. Medical treatments (e.g., tamoxifen, bromocriptine) showed variable outcomes, with most patients exhibiting no hormonal abnormalities.

**Conclusions:**

Among breast cancer treatments subcutaneous mastectomy stands as the most successful method that results in few recurrences. Documentation of psychological distress created by VBH demands collaborative care strategies and extensive investigations into genetic molecular influences.

## Introduction

Virginal Breast Hypertrophy (VBH), a myriad of terms describing this entity in the literature including Juvenile Breast Hypertrophy, Juvenile Gigantomastia, and Juvenile Macromastia^[1,2]^, is a benign condition where atypical, alarmingly rapid, and continued breast growth occurs during puberty. It often follows a 6-month period of extreme breast enlargement, superseded by a longer period of slower but sustained breast growth^[3]^ causing discomfort, pain, and postural and movement disturbance. This happens even though one’s estrogen levels are normal. It is aggravated by high sensitivity of estrogen receptors in the breast or a shift in the estrogen/progesterone balance^[4,5]^. VBH can be present at birth, or develop during puberty. In rare instances, the condition begins in a prepubertal or peripubertal young woman with no prior experience of breast development, still, the exact causes of VBH are uncertain and include hormonal factors, inheritance, and others^[4,6]^. This condition exposes female adolescents to severe psychosocial stressors resulting from the growing up process especially the development of the wrong perception towards the body shape. Psychological manifestations include depressions, eating disorders concerning body image, and poor self-image^[7,8]^. Some of the physical symptoms include skin redness, dilated subcutaneous tissue, and even skin necrosis according to the rates of the growth^[5,9-11]^. The differentiated diagnosis of hyperplastic breast anomalies in adolescents also includes pregnancy, fibrocytosis, adolescent gigantomastia, VBH, and tumors in the form of juvenile fibroadenoma, phyllodes tumor, juvenile papillomatosis, and increased endogenous or exogenous hormonal levels. While one can confidently say that malignant tumors are extremely rare in this population^[12-15]^, they should still be considered when there is breast asymmetry. The contemporary obesity pandemic has made the differentiation of adolescents with macromastia from those suffering from breast hypertrophy caused by obesity even more difficult^[16]^.

**Highlights**

- A systematic review of 69 VBH cases was conducted, focusing on patient characteristics, clinical manifestations, and treatment outcomes.
- The average onset duration for VBH development was found to be 10 months, with a mean patient age of 11.27 years.
- Surgical treatment, particularly subcutaneous mastectomy, showed higher effectiveness compared to medical treatment options.
- Recurrence of VBH post-treatment was low for surgical approaches but varied with medical interventions.
- Psychological impacts and the amount of tissue removed during surgery were key factors analyzed in determining treatment success.
- The study also evaluated menarche status and its correlation with disease progression.

Treatment options for VBH have evolved over time, ranging from conservative approaches to surgical interventions. Conservative methods may include lifestyle modifications, supportive bras, and pain management techniques. Surgical options often involve reduction mammoplasty, which aims to reduce breast size and alleviate symptoms. The most challenging aspect in the management of VHB is the difficulty in effecting a definitive treatment. The relentless course of this disease and the refractory nature to surgery is well documented^[1-3]^. Surgical treatment with reduction mammaplasty is ideal but will almost invariably result in recurrence, necessitating secondary reduction or mastectomy.

VBH has profound psychosocial consequences for affected adolescents. The rapid changes in body shape and size during a crucial developmental period lead to heightened self-consciousness and social anxiety. Adolescents with VBH often experience significant psychological stress, including depression, eating disorders related to body image, and poor self-esteem. The physical symptoms, such as skin redness, dilated subcutaneous veins, and potential skin necrosis, further exacerbate the psychological burden, as these visible changes can lead to social stigma and isolation. Healthcare providers must be aware of the psychosocial challenges associated with VBH and offer appropriate interventions to address issues, including management of the condition, providing counseling, and offering mental health support to help patients cope with the emotional and psychological impacts. As the etiology of VBH is multifactorial-hormonal, genetic, and environmental-in the management of this issue, a multidisciplinary diagnosis and treatment approach is required that attends to both somatic and psycho-social aspects of the patient’s condition.

This review focuses on VBH in adolescents, emphasizing the psychosocial and clinical issues that are frequently overlooked in the available literature. Unlike previous studies, it emphasizes the condition’s hormonal sensitivity, diagnostic complexity, and high surgical recurrence rates, providing insights into the necessity for a personalized, interdisciplinary approach to better therapeutic techniques.

## Methodology

The Preferred Reporting Items for Systematic Reviews and Meta-Analyses (PRISMA) guidelines were followed in the development of this systematic review of the literature^[17]^. We aimed to respond to the following research question: What are the characteristic clinical features, diagnostic patterns, treatment options, effectiveness, recurrence rates, and psychosocial impacts of VBH and what gaps exist in the current understanding of this condition?

The study population consisted of adolescent women, whose intervention of interest was breast hypertrophy; the comparator in studies with a control group was adolescents with no history of breast hypertrophy. Only case reports that offer comprehensive clinical descriptions and specific case outcomes are the only ones taken into account. Studies produced in languages other than English or with participants outside of the adolescent age range are also not included. Review articles, meta-analyses, and systematic reviews were excluded, as are research that concentrate on other breast disorders such fibroadenoma or gestational macromastia. Meta-analyses and systematic reviews are not included because they provide broad conclusions and may not capture the detailed clinical presentations and outcomes of individual individuals, which are critical to this investigation. Furthermore, these reviews frequently involve heterogeneous data or broader scopes, addressing breast illnesses outside than the study’s emphasis. The study’s prioritization of thorough case reports ensures a more precise investigation of virginal breast enlargement in adolescents.

A literature search was conducted in August and September 2024 in the medical databases PubMed (Medline), Embase and Medline. We analyzed all case reports on VBH from 1934 to 2023.We used the search strategy as (“virginal breast hypertrophy “OR” juvenile breast enlargement “OR” juvenile macromastia “OR” juvenile gigantomastia”) AND (treatment OR management) AND (case report) and Boolean operators and MeSH terms where applicable, Duplicate studies were removed. A minimum of two reviewers reached a consensus on the inclusion of studies and the selection of eligible studies based on their independent agreement. This review did not follow any specific protocol.

The search was carried out in pairs, with two authors (ES and MK) who independently selected the titles of the articles and then proceeded to read the abstracts and full texts. The following information was extracted using a model created by the study’s authors: author(s), year, population and sample, age, clinical breast hypertrophy characterization, related symptoms and complications, diagnostic techniques, and treatment. Primary outcomes were treatment options, effectiveness, recurrence rates and psychological impact.

Although our study predominantly focuses on the adolescent population, some cases include patients aged 18, 20, and 22. These individuals were included because they developed VBH during their adolescence but were reported in case studies later in life at the aforementioned ages. This inclusion aligns with our diagnostic criteria, which prioritize the timing of symptom onset rather than the age at which the case was documented, ensuring a comprehensive understanding of VBH across its natural history.

In all patients, age at diagnosis and duration of symptoms before therapy are recorded as part of the patient demographics analysis. In order to look at possible genetic factors and correlations with the development of symptoms, the study also assessed menarche status (reached or not reached) and family history (yes or no) of breast cancer. Procedures involving surgery, such as subcutaneous mastectomy or reduction mammoplasty, the quantity of tissue removed, their efficacy, and any recurrence (recurrence or no recurrence), are all recorded. This review also included the medical interventions (bromocriptine, tamoxifen, dydrogesterone, medroxyprogesterone, chorionic gonadotropic hormone, estrogenic substance, thyroid extract, oral NSAID) that have been used, evaluate their efficacy based on patient-reported results, and record any side effects, such as axillary lymph node tumors and psychological impacts (yes or no). The results of the breast ultrasonography were also analyzed for anomalies, and progesterone, FSH, LH, estradiol, prolactin, TSH, and prolactin levels were evaluated to detect any hormonal imbalances linked to VBH (normal or elevated).

When assessing the quality of case studies, the format suggested by Murad *et al*^[18]^ was used, which consisted of four domains: selection, verification, causality, and communication. A general judgment on methodological quality was made according to the recommendations; all studies were of satisfactory quality (Table 1).

**Table 1:** Evaluation of the methodological quality of the articles included in this review

Domain	Explanatory questions	Methodological quality
Selection	Does the patient(s) represent(s) the whole experience of the investigator (center) or is the selection method unclear to the extent that other patients with similar presentation may not have been reported?	Yes
Ascertainment	Was the exposure adequately ascertained?	Yes
	Was the outcome adequately ascertained?	Yes
Causality	Were there other alternative causes that may explain why the observation was ruled out?	Yes
	Was there a challenge/rechallenge phenomenon?	No
	Was there a dose–response effect?	No
	Was follow-up long enough for outcomes to occur?	Yes
Reporting	Is the case(s) described with sufficient details to allow other investigators to replicate the research or to allow practitioners to make inferences related to their own practice?	Yes

**Figure 1.**
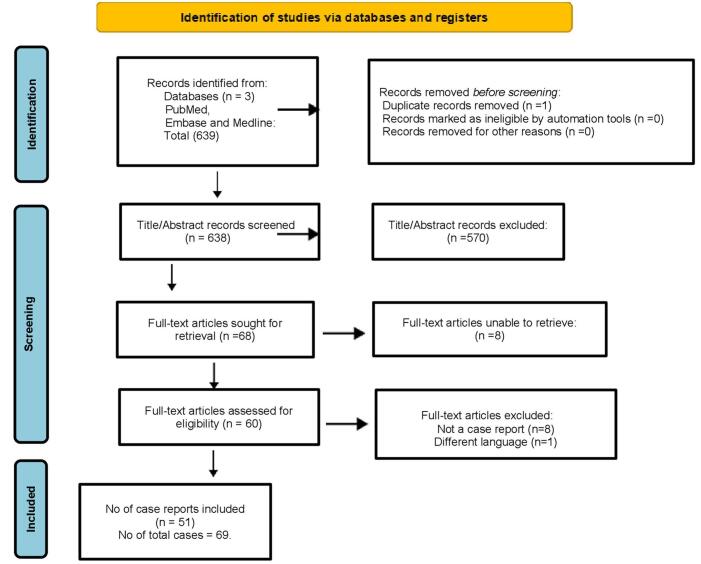
PRISMA flow chart.

The PRISMA flow chart (Fig. 1) describes the methodical procedure for finding, vetting, and choosing research to be included in a review. Three databases (PubMed, Embase, and Medline) yielded a total of 639 records at first. A total of 638 records were screened using titles and abstracts after one duplicate was eliminated; 570 records were eliminated at this stage. There are 60 full-text articles awaiting eligibility determination out of the 68 that were requested for retrieval; 8 could not be obtained. Finally 51 case reports analyzed after removing 9 studies among which 8 studies were not case reports and 1 study has a different language than English.

## Results:

In this systematic review of VBH, the findings are organized from patient characteristics through to treatment outcomes. The review encompasses data from 69 patients, revealing a mean duration for disease development of 10 months and a mean age of approximately 11.27 years. Menarche had been reached in 43.75% of patients, while 56.25% had not yet reached menarche. The most common clinical manifestations included rapid, painful breast enlargement in all cases, with bilateral involvement observed in 91.3% of patients (see Table 2). Progesterone, FSH, LH, estradiol, prolactin, TSH, and prolactin levels were also evaluated to detect any hormonal imbalances linked to VBH but found to remain normal in almost all cases, only five cases reported elevated prolactin levels.

**Table 2:** Summary of clinical characteristics of virginal breast hypertrophy cases

Characteristics (*n* = 69)	Number of patients
Duration of disease development in months, mean ± S.D: (*n* = 29)	10 (11.70)
Menarche status (*n* = 47):	
-Reached	43.75%
-Not reached	56.25%
Signs and symptoms: (*n* = 69)	
-Rapid painful breast enlargement	100%
-Bilateral	91.3%
-Unilateral	8.69%
-Erythema and edema	30%
-painless enlargement	35%
-dilated superficial vessels	30%
-posture issue and backpain	25%
-Striae	8%
-Skin changes	32.5
Psychological issues	71%
Any known co-morbidity	7%
Any medication history	4%

**Figure 2.**
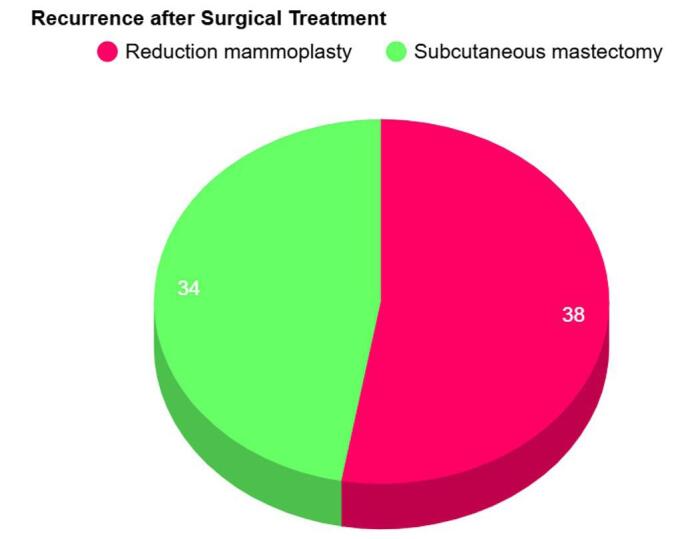
Pie chart of recurrence among patients after surgical treatment.

Surgical management played a crucial role in treatment. Reduction mammoplasty was performed in 38 cases, resulting in a recurrence rate of 44.7%. In contrast, subcutaneous mastectomy, undertaken in 34 cases, demonstrated a significantly lower recurrence rate of 5.8% (Fig. 2). The mean amount of tissue removed during surgery was 6679.64 grams, with a standard deviation of 2772.57 grams. Regarding medical treatments, tamoxifen effectively reduced breast bulk and halted growth, with patients showing negative estrogen receptor status. Dydrogesterone, however, was associated with continued breast growth and did not manage the condition effectively. Bromocriptine succeeded in lowering prolactin levels but did not lead to a reduction in breast size. Other medications, such as Oral NSAIDs, Medroxyprogesterone, Danazol, Chorionic Gonadotropic Hormone, and Thyroid Extract were reported but lacked specific outcome data. (see Table 3). Only four cases were found to have family history of breast disease, while the majority had no family history of breast disease (see Table 4).

**Table 3:** Management approaches and outcomes for virginal breast hypertrophy

Surgical management	*n*	Recurrence rate
Reduction mammoplasty	38	44.7%
Subcutaneous mastectomy	34	5.8%
Amount of total tissue removed		
6679.64 (2772.57) grams		
Mean (SD)		
Medical treatment reported:		Outcomes
Tamoxifen		Reduced breast bulk, growth and negative estrogen receptor
Dydrogesterone		Continued to experience breast growth
Oral NSAID		
Medroxyprogesterone		
Bromocriptine		Lowered prolactin but initial enlargement persisted
Danazol		
Chorionic gonadotropic hormone		
Thyroid extract

**Table 4:** Case report summary: study names, age, and family history of breast disease

Studies	Age (years)	Family hx of breast disease	Quality assessment^[19]^
Sarah A Soliman, 2023^[19]^	11	None	Good
Patricia M O Hare, 2000^[20]^	14	None	Good
^	12	None	Good
G Ağaoğlu, 2000^[21]^	16	None	Good
R F Ryan, 1985^[22]^	19	NR	Good
R Samuelov, 1988^[23]^	10	NR	Good
Gülay Karagüzel^[24]^	12.5	None	Good
^	11.5	None	Good
Jacky Govrin-Yehudain, 2004^[25]^	17	Familial VBH	Good
^	13	Familial VBH	Good
^	14	Familial VBH	Good
^	12	Familial VBH	Good
ILDI H Koves, 2007^[26]^	11	None	Good
Ebru Menekse, 2014^[27]^	12	None	Good
Edyta Szymańska, 2018^[28]^	11	None	Good
Biraj Pokhrel, 2020^[29]^	14	None	Good
D T Netscher, 1996^[30]^	13	NR	Good
Tarek Ewies, 2013^[31]^	11	NR	Good
P. sagot, 1990^[32]^	11.5	None	Good
Akmal Hisham, 2017^[4]^	13	None	Good
Korcan Demir, 2010^[33]^	12.5	None	Good
Linda Fiumara, 2009^[34]^	12	None	Good
D W Furnas, 1982^[35]^	12	NR	Good
G. D. L. Arscott, 2001^[36]^	12	None	Good
C Cardoso, 1990^[37]^	16	NR	Good
T Morimoto, 1993^[38]^	12	None	Good
F Gentimi, 2011^[39]^	12	None	Good
Oliver Schumacher, 2009^[40]^	12	None	Good
Abakka Sanae, 2013^[41]^	14	NR	Good
Gottfried Wechselberger, 2004^[42]^	15	Mother had breast cancer	Good
Henry Albert, M.D, 1910^[43]^	13	None	Good
Steven W, Boyce, 1984^[44]^	24	NR	Good
Cardoso de Castro M.D, 1990^[37]^	16	None	Good
Joseph A. Gaines, M.D., 1937^[45]^	14	None	Good
James B. Gillespie, M.D, 1949^[46]^	12	NR	Good
B. A. Goodman, M.D., 1934^[47]^	14	NR	Good
A. Gliosci, M.D, 1993^[48]^	14	NR	Good
^	14	NR	Good
^	20	NR	Good
^	15	NR	Good
A A Al Saif, 2009^[49]^	12	None	Good
Wilkins, 1965^[50]^	10	NR	Good
Wakeley C. 1934^[51]^	10	NR	Good
GR Sridhar, 1995^[52]^	12	None	Good
^	18	None	Good
RL Sperling, 1973^[53]^	12	None	Good
AG Ship, 1971^[10]^	16	None	Good
p pulzl, 2005^[54]^	21	None	Good
H A Oberman, 1979^[55]^	11	NR	Good
^	13	NR	Good
^	17	NR	Good
Mayl *et al*, 1974^[56]^	11	NR	Good
^	20	NR	Good
^	21	NR	Good
^	18	NR	Good
D kupfer, 1992^[9]^	11	NR	Good
^	12	NR	Good
A. Khan, 2000^[57]^	15	None	Good
Hollingsworth and Archer, 1973^[58]^	14	NR	Good
Harris and Rosenthal, 1945^[59]^	15	NR	Good
Baker *et al*, 2001^[5]^	17	NR	Good
^	10	NR	Good
^	11	NR	Good
De Castro, 1977^[60]^	12	NR	Good
Erich, 1960^[61]^	13	NR	Good
Fisher and Smith,1971^[62]^	10	NR	Good
^	18	NR	Good
Fisher *et al*, 1943^[63]^	11	NR	Good
Gerber, 2004^[64]^	13	NR	Good

Hx = history, NR = not reported.

Overall, surgical options provided significant tissue reduction and lower recurrence rates, while medical treatments varied in effectiveness, with some offering symptomatic relief but failing to address the underlying growth of breast tissue. This review highlights the need for tailored treatment approaches to address both the physical and psychological aspects of VBH.

## Summary

This review involved 69 patients with VBH and found that most patients experienced rapid, painful breast enlargement, with bilateral involvement observed in 91.3% of cases. Surgical management was crucial in treatment, with reduction mammoplasty in 38 cases and subcutaneous mastectomy in 34 cases resulting in lower recurrence rates. Medical treatments included tamoxifen, dydrogesterone, bromocriptine, and other medications. The majority of cases had no family history of breast disease, and the review emphasizes the need for tailored treatment approaches to address both physical and psychological aspects of VBH.

## Discussion

VBH remains a challenging clinical entity, with its characteristic rapid breast enlargement in adolescent girls posing significant physical and psychological burdens. Our systematic review aimed to answer critical questions about the clinical features, diagnostic approaches, treatment options, recurrence rates, and psychosocial impacts of VBH, while also identifying gaps in the current literature.

The characteristic clinical manifestation of VBH, as noted in this review, is alarmingly rapid bilateral breast enlargement, which occurred in 91.3% of patients, consistent with previous reports that highlight VBH as a bilateral process affecting most adolescents^[3]^. However very few unilateral cases of VBH are also reported^[21,32,33,48,54]^. Psychological and emotional issues, including depression and low self-esteem, were reported in 71% of patients, further underscoring the condition’s impact on mental health, body image, and social functioning. These findings are in line with existing research suggesting that the psychological toll of VBH is often more profound in adolescent patients than in adults due to heightened social pressures to conform to idealized body norms^[65]^. In cases of VBH, the most prominent symptom is rapid and progressive breast enlargement, often bilateral, which can lead to significant discomfort and complications^[7,8]^. Many patients experience skin stretching, resulting in striae, erythema, edema, and in severe cases, ulceration and peau d’orange^[27]^. Additional symptoms include dilated superficial veins, shoulder grooving, and postural changes such as kyphosis, slouched posture, and lordosis, particularly in cases where the breast enlargement is massive^[10,23]^. Patients may also report back and shoulder pain, a dragging sensation in the chest, and in some instances, inverted nipples and galactorrhea^[38]^. Despite the significant enlargement, some cases remain painless^[19]^, though many patients experience mastalgia (breast pain) and tenderness, often associated with edema and erythema These symptoms not only cause physical discomfort but also lead to psychological distress, impacting body image and self-esteem. The skin’s quality can degrade over time due to hyper distension, contributing to pressure sores and other skin abnormalities^[20]^. Palpation findings vary widely, reflecting the diverse presentations of the condition. In many cases, no palpable masses or nipple discharge are noted, although some patients may exhibit tenderness, generalized nodularity, or firm masses^[20]^. Others may present with large, firm, and poorly defined masses. Ultrasound and imaging often reveal fibroglandular tissue, cystic masses, or homogeneous masses without signs of malignancy^[66]^. These varying palpation findings are crucial in guiding further diagnostic and treatment approaches, emphasizing the complexity and heterogeneity of VBH presentations.

Differential diagnosis remains complex due to the overlap of clinical features with other breast pathologies such as fibroadenomas, phyllodes tumors, and hormonal imbalances^[12-15]^. Imaging techniques such as ultrasound and MRI are commonly used to rule out these other causes, but the dense breast tissue in adolescent girls complicates mammographic assessments^[67]^. Histological findings in VBH, including Fibroadenomatous Changes, glandular or ductal epithelial proliferation, dense fibrotic stroma, and the absence of Atypia, help further distinguish VBH from malignant breast tumors, which are exceedingly rare in this population^[14,15]^, however any breast surgical resection specimen should be routinely subjected to histopathological examination due to the low but possible chances of malignancy.

The exact etiology of VBH remains elusive. Our review supports Griffith’s estrogen hypersensitivity theory^[11]^, which suggests that breast tissue in VBH patients is more responsive to normal estrogen levels, leading to excessive growth. As research findings indicate enhanced estrogen receptor activity in resected breast tissues, this aligns best with the estrogen hypersensitivity theory, suggesting that localized estrogen production or sensitivity may play a pivotal role in VBH development. While phosphatase and tensin homolog (PTEN) mutations and Cowden syndrome have been implicated in some familial cases of VBH^[68,69]^. Though the PTEN gene mutation analysis was done in pathological samples of two case reports, the authors found the negative results^[24,26]^, further research is needed to establish a stronger genetic link, as current evidence from mutation analysis has been inconclusive.

Treatment strategies also reflect established surgical and medical guidelines, with subcutaneous mastectomy recommended as the most effective approach, in line with current surgical practices for macromastia, while reduction mammoplasty remains a viable option for those seeking breast preservation, despite its higher recurrence rates. Surgical interventions, particularly subcutaneous mastectomy, with our review showing a low recurrence rate of 5.8%, compared to 44.7% for reduction mammoplasty. This is consistent with previous literature indicating that subcutaneous mastectomy offers the highest likelihood of preventing recurrence^[2,70]^. However, surgery is not without its drawbacks. Timing of the surgery is critical, as premature intervention may result in the need for additional operations due to continued breast growth. Reduction mammoplasty, while providing immediate relief from symptoms, carries a higher risk of recurrence and may necessitate further surgical interventions. Medical treatments, such as tamoxifen and bromocriptine, showed varying levels of effectiveness. Tamoxifen was found to halt breast growth in estrogen receptor-negative patients^[24]^, but dydrogesterone and bromocriptine did not significantly reduce breast size^[36,44,54]^, reflecting the unpredictable efficacy of non-surgical treatments. Other medical treatments that have been used in different studies include oral NSAIDs, Medroxyprogesterone, Danazol, Chorionic Gonadotropic Hormone, and Thyroid Extract were reported but lacked specific outcome data^[27,44,52]^. These findings highlight the need for more targeted therapies based on hormonal receptor status, as medical interventions currently offer symptomatic relief but fail to address the underlying cause of breast tissue overgrowth

Recurrence rates were significantly lower with subcutaneous mastectomy compared to reduction mammoplasty, reinforcing the importance of choosing the appropriate surgical method to reduce the risk of reoperation. However, in younger patients, the risk of psychological distress from repeated surgeries must be carefully weighed against the benefits of tissue reduction. This points to a critical gap in the literature: the lack of long-term follow-up studies on post-surgical outcomes, both in terms of physical recurrence and psychosocial well-being. Further research is needed to establish long-term recurrence rates and the psychological impact of different treatment modalities over time.

Adolescents are significantly impacted psychologically by VBH, hence a multidisciplinary strategy that includes psychologists is crucial^[71]^.The psychosocial impacts of VBH cannot be overstated. As reported in our review, 71% of patients experienced significant emotional distress, body image issues, and low self-esteem, which are common in adolescent patients with VBH. Depression, eating disorders, and social withdrawal are frequently observed in this patient population. While surgical intervention can alleviate physical symptoms, it does not directly address the psychological issues, highlighting the need for multidisciplinary care that includes psychological support as part of the treatment plan.

Over the 13 years since Hoppe *et al*’s^[2]^ analysis of VBH, significant parallels and advancements in understanding this condition have emerged. Hoppe’s review of 65 cases highlighted estrogen receptor hypersensitivity and potential genetic factors, such as PTEN gene mutations, as key contributors. Similarly, our review of 69 cases supports receptor hypersensitivity as a central mechanism but refines the understanding by exclusively focusing on cases of VBH, excluding gestational and other secondary causes that were included in Hoppe’s dataset. This approach not only ensures a more specific analysis but also incorporates eight new cases, offering updated findings on hormonal receptor involvement and reaffirming the limited role of genetic mutations like PTEN.

In terms of treatment, Hoppe emphasized the recurrence of breast growth following reduction mammoplasty, recommending subcutaneous mastectomy for more definitive outcomes. Our findings corroborate this recommendation while reflecting broader adoption of less deforming surgical techniques and advances in medical management, such as the use of tamoxifen and newer hormonal agents, despite their side effects. Both analyses underscore the psychological impact of VBH and the importance of early diagnosis; however, our review expands on this by addressing the need for enhanced mental health support and patient education, particularly for adolescents. Overall, while Hoppe’s work laid a strong foundation, our findings provide a more focused and updated perspective, incorporating new cases and evolving clinical insights.

Longitudinal cohort studies would involve observing adolescents with VBH for 10–15 years in terms of psychosocial outcomes by employing standardized tools like Rosenberg Self-Esteem Scale and Quality of Life assessment. It will include deep qualitative interviews into the personal experience as well as on how VBH may influence the child’s social-emotional development. Another aspect through which effectiveness in the intervention process can be explored for psychological support treatments like CBT would be regarding improvement in the outcome of mental health. Identification of genetic markers is critical through studies on genome-wide association and PTEN mutation to be used for VBH diagnosis. Hormonal profiling, specifically on estrogen receptors, in the context of breast tissue may shed further light into the hormonal basis of VBH. Clinical studies that will involve hormonal therapies, such as tamoxifen, will determine whether such treatments will effectively treat VBH. Together, all these will ultimately culminate in tailored treatments and better long-term outcomes for the patients who are diagnosed with VBH.

Future studies should concentrate on integrating findings into clinical practice by creating standardized diagnostic criteria and treatment regimens that incorporate hormone receptor profiling, genetic testing, and psychological evaluation. A multidisciplinary strategy that includes surgeons, endocrinologists, and psychologists can address both physical and emotional health concerns. Clear standards and long-term follow-up will guarantee that VBH patients receive tailored, evidence-based care and achieve better outcomes.

## Limitations

Despite advancements in understanding the clinical and surgical management of VBH, significant gaps remain. The precise etiology of VBH, especially its hormonal underpinnings, is still not fully understood. Further research into genetic factors such as PTEN mutations and estrogen receptor activity is essential to developing more targeted therapies. Additionally, the variability in response to medical treatments points to a need for better biomarkers to predict treatment success and recurrence. Another gap is the lack of standardized diagnostic criteria and long-term outcome data. Current diagnostic approaches rely on exclusion rather than definitive markers for VBH, making early diagnosis and intervention more difficult. More longitudinal studies are needed to assess the psychosocial outcomes and long-term recurrence rates following different treatments, as well as to explore potential preventive strategies for at-risk populations.

## Conclusion

VBH is an uncommon but serious illness in teenagers that causes fast and typically bilateral breast expansion, posing severe physical and mental issues. This review focuses on the hormonal sensitivity, diagnostic challenges, and high recurrence rates associated with VBH, particularly after surgical procedures. The outcome of this literature review highlights the demand for thorough and integrated management by doctors regarding VBH in adolescents. The clinician would have to analyze both physical and psychological impacts while making a decision on the type of treatment plan to be followed. A strongly reduced recurrence rate of subcutaneous mastectomy as compared to that with reduction mammoplasty is recommended for surgical decisions, especially in teens, who experience more psychological impacts from multiple surgery sessions. The serious psychological consequences of VBH, such as depression, body image difficulties, and social disengagement, highlight the importance of a multidisciplinary strategy that combines physical treatment with mental health assistance. Treatment planning must incorporate mental health support interventions to face the high prevalence of psychological problems associated with VBH. Tailored treatment plans according to hormonal receptor status and adequate continuous long-term follow-up will be necessary for better patient outcomes. Thus, Subcutaneous mastectomy remains the most effective surgical option, with medicinal therapies having limited and inconsistent efficacy. More research is needed to improve diagnostic methods, treatment options, and long-term results for adolescents with this illness.

## Data Availability

The datasets used and/or analyzed during the current study are available from the corresponding author upon reasonable request.

## Registration of research studies

PROSPERO (CRD42024596587).
